# A Fiber-Optic Non-Invasive Swallowing Assessment Device Based on a Wearable Pressure Sensor

**DOI:** 10.3390/s23042355

**Published:** 2023-02-20

**Authors:** Masanori Maeda, Miyuki Kadokura, Ryoko Aoki, Noriko Komatsu, Masaru Kawakami, Yuya Koyama, Kazuhiro Watanabe, Michiko Nishiyama

**Affiliations:** 1 Information Systems Science Major, Graduate of Science and Engineering, Soka University, 1-236 Tangi-Machi, Hachioji, Tokyo 192-8577, Japan; 2 Faculty of Nursing, Soka University, 1-236 Tangi-Machi, Hachioji, Tokyo 192-8577, Japan; 3 School of Nursing, Jichi Medical University, 3311-159 Yakushiji, Shimotsuke, Tochigi 329-0431, Japan; 4 Department of Electrical and Electronic Engineering, Chiba Institute of Technology, 2-17-1 Tsudanuma, Narashino, Chiba 275-0016, Japan; 5 Department of Science and Engineering for Sustainable Innovation, Faculty of Science and Engineering, Soka University, 1-236 Tangi-Machi, Hachioji, Tokyo 192-8577, Japan

**Keywords:** health monitoring, swallowing function test, dysphagia, hetero-core fiber-optic sensor, non-invasive system, wearable

## Abstract

We developed a wearable swallowing assessment device using a hetero-core fiber-optic pressure sensor for the detection of laryngeal movement during swallowing. The proposed pressure sensor (comfortably attached to the skin of the neck) demonstrated a high sensitivity of 0.592 dB/kPa and a linearity of R^2^ = 0.995 within a 14 kPa pressure band, which is a suitable pressure for the detection of laryngeal movement. In addition, since the fabricated hetero-core fiber-optic pressure sensor maintains appreciable sensitivity over the surface of the sensor, the proposed wearable swallowing assessment device can accurately track the subtle pressure changes induced by laryngeal movements during the swallowing process. Sixteen male subjects and one female subject were evaluated in a variety of age groups ranging from 30 to 60 years old. For all subjects, characteristic swallowing waveforms (with two valleys based on laryngeal movements consisting of upward, forward, backward, and downward displacements) were acquired using the proposed wearable swallowing assessment device. Since the denoted time of the first valley in the acquired waveform determines the “aging effect”, significant differences in swallowing functions among the different age groups were ultimately determined based on the time of the first valley. Additionally, by analyzing each age group using the proposed device, due to *p*-values being consistently less than 0.05, swallowing times were found to exhibit statistically significant differences within the same groups.

## 1. Introduction

Recently, an increase in the proportion of the elderly as a percentage of total population has been occurring worldwide. Researchers predicted that, by the year 2050, half of all countries and regions will soon be categorized as “aging societies” and 15% will be “super-aging societies” [1,2,3]. Population aging is causing various social problems, such as increased social security costs and burdens on caregivers. Extending the period during which a person can live healthily improves the person’s well-being. It reduces burdens on caregivers, in addition to being socially and economically meaningful [3]. For an extended life expectancy, detecting the early decline in biological functions through routine monitoring at home is desirable.

Among the biological functions that decline with aging is the swallowing function. Dysphagia, caused by an age-related decline in the swallowing function, reduces quality of life [4,5,6]. Moreover, it leads to the manifestation of aspiration pneumonia, which is a leading cause of death in the elderly [5,7]. A decrease in the swallowing function has been confirmed by measuring the elevation extension time of the laryngeal prominence during swallowing [8]. The swallowing time assessment is known to be a practical measurement of swallowing dysfunction [9]. Therefore, a more accurate measurement of swallowing time can contribute to the measurement of swallowing dysfunction. Furthermore, during the swallowing function rehabilitation process, speech and language pathologists regularly confirm the position of laryngeal prominence from the skin of the neck, and they are trained to increase the elevation of the laryngeal prominence [10]. In such cases, non-invasively detecting the position of the laryngeal prominence is desirable because the overall effectiveness of swallowing function training is evaluated by measuring the position of laryngeal prominence.

A videofluoroscopy swallowing study (VFSS) is a well-known swallowing function test performed in clinical practice [11,12]. Although this method can directly examine the swallowing function, it is considered invasive due to the required radiation exposure of the patient. Additionally, it imposes the burden of hospital visits on the patient because the examination site is limited to a medical institution [11]. A videoendoscopic swallowing study (VESS) [12,13], in contrast, is a bedside procedure; however, examiners are strictly limited to certified healthcare professionals. Therefore, such studies are not suitable for the routine monitoring of the swallowing function because patients typically desire that such methods be non-invasive, simplistic, and not determined by location.

Researchers proposed numerous swallowing function sensing techniques, which patients can easily use at bedside [14,15,16,17,18]: for example, sensing methods detecting sounds produced during swallowing [14,15] as well as methods that directly detect movement of the laryngeal prominence [16,17,18]. Further, detecting sounds produced during swallowing, measuring the time taken to raise the laryngeal prominence is also possible. However, detecting the exact position of the prominence based on sound detection alone is difficult. Various techniques for measuring the movement of the laryngeal prominence, such as an electric resistance-based bend sensor [16], a thick film polymer pressure sensor [17], and a vision-based method [18], have all been developed as non-invasive approaches. The bend sensor can detect laryngeal movement by attaching it to the larynx; however, it entails the risk of skin tears during the removal process because elderly skin is often fragile. The thick film polymer pressure sensor can detect laryngeal movement without attaching it directly to the skin at the larynx. However, it consists of metal wires such as electrical resistive elements, and the current flows directly to the sensor part; therefore, electromagnetic noise can affect them. The vision-based method of using a charge-coupled device (CCD) camera measures laryngeal elevation from captured images. This method often affects measurement accuracy due to a strong dependence on the pictorial clarity of the subject’s laryngeal prominence.

In response to the various shortcomings discussed above, we proposed a hetero-core optical fiber bending sensor as a viable and practical alternative for swallowing health care [19,20,21]. Compared to other optical fiber bending sensors, hetero-core optical fiber bending sensors are cost-effective because they can measure bending properties using LEDs with a light intensity basis. In addition, such sensors do not require temperature compensation; therefore, the measurement system can be constructed as a simple configuration. Furthermore, the sensor itself does not require a power supply. The diminutive characteristics of fiber optics, such as light mass and small diameter, also allow the weight and size of the sensor to be reduced [22]. A hetero-core fiber bend sensor was fabricated using a single-mode optical fiber made of silica glass; although it is possible to fabricate a hetero-core structure using POF or multimode optical fibers, the use of a single-mode optical fiber made of silica glass makes the sensor less susceptible to changes in transmission line curvature [23]. The fiber is less susceptible to changes in the curvature of the transmission line.

The laryngeal elevation during the end of the oral phase and during the pharyngeal phase of swallowing induces a pressure change upon the skin surface of the neck. Our team previously successfully developed pressure sensor hetero-core fiber-optic technologies for other biomedical applications such as that for respiration monitoring [19] and for the monitoring of walking posture (with sensors embedded into insoles [20]). Fixing such a pressure sensor directly onto the larynx eliminates the need to stick it onto the skin, thus eliminating the potential for skin tears. Increasing the sensitivity of the hetero-core fiber-optic pressure sensor to pressure changes incurred during laryngeal movement allows subjects without a distinct laryngeal prominence, such as women, to successfully utilize the sensor.

In this manuscript, we hence propose the potential deployment of a wearable swallowing assessment device using a hetero-core fiber-optic pressure sensor for detecting laryngeal movement during patient swallowing evaluations. To reduce possible overload to the larynx, a soft puff is attached to the sensor. Accordingly, due to the inserted puff, the sensor needs to maintain a high sensitivity to low pressures. Additionally, to implement it for users without a distinct laryngeal prominence, such as women, we devised a distinct pressure sensor design that ultimately enhances its overall sensitivity. In order for the pressure sensor to be fixed at a specific position to accurately trace the movement of laryngeal prominence, the sensor was set on a dome-shaped base in order to maximize displacement flexibility. The fiber-optic pressure sensor developed for laryngeal movement detection exhibited various sensing properties, including a sensitivity value of 0.592 dB/kPa and an R-square value of 0.995 when the sensor was pressurized and a sensitivity value of 0.606 dB/kPa and an R-square value of 0.997 when the sensor was pressurized for linear fitting, along with a 7.40% degree of hysteresis. The force-sensing resistor (FSR)-based polymer pressure sensor, as the typical commercial sensor, has a 10% degree of hysteresis [24], comparable to the proposed fiber pressure sensor. Although the thickness of the FSR sensor of less than 1 mm is thicker than the proposed fiber-optic pressure sensor, the proposed fiber-optic pressure sensor can reduce the thickness because the fiber optics has a thin size with a diameter of 250 um, including the protective coating. Next, we evaluated the proposed wearable swallowing assessment device for 16 male subjects ranging from 30 to 60 years old, along with one female subject in her 20s. The proposed wearable swallowing assessment device successfully picked up the typical swallowing temporal waveform for all the subjects. A one-way analysis of variance was performed for each subject within each group to analyze whether significant differences were present in swallowing times for each age group. The results ultimately show that notable differences were indeed present in swallowing times among subjects within all age groups.

## 2. Materials and Methods

### 2.1. A Hetero-Core Fiber-Optic Pressure Sensor

**Figure 1:**
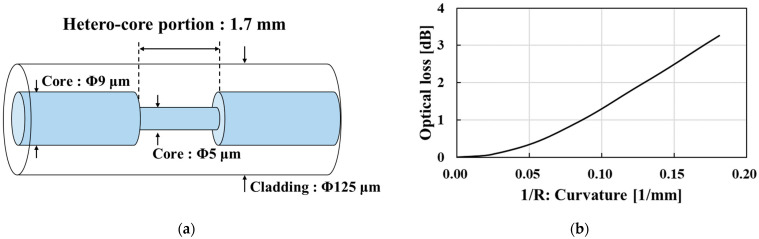
Hetero-core fiber-optic bending sensor: (**a**) sensor structure; (**b**) bending curvature characteristics in the optical loss change.

Figure 1 shows the structure of a hetero-core fiber-optic bending sensor and its bending curvature characteristics in the optical loss change. The hetero-core fiber-optic bending sensor consists of a 9 μm core single-mode transmission fiber and a 5 μm fiber with a length of 1.7 mm, inserted using fusion splicing, as shown in Figure 1a. In our previous works, researchers established that hetero-core fiber-optic sensor technology exhibits high optical loss monotonic sensitivity to the bending of the hetero-core portion, allowing the bending of the hetero-core portion to be accurately measured on an optical intensity basis [22]. Next, the hetero-core fiber bending sensor exhibits a trade-off property, however, between bending sensitivity and linearity, depending on the length of the hetero-core portion within the range of 1–2 mm at a wavelength of 1.31 μm. Further, we employed a hetero-core inserted length of 1.7 mm because a hetero-core fiber sensor with a 1.7 mm length demonstrates a notably high sensitivity relative to its linearity [20,22].

**Figure 2:**
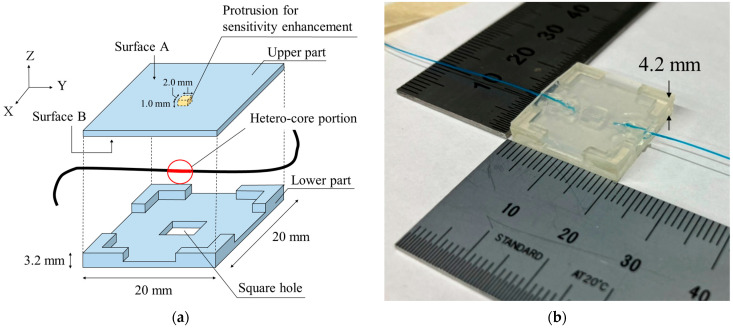

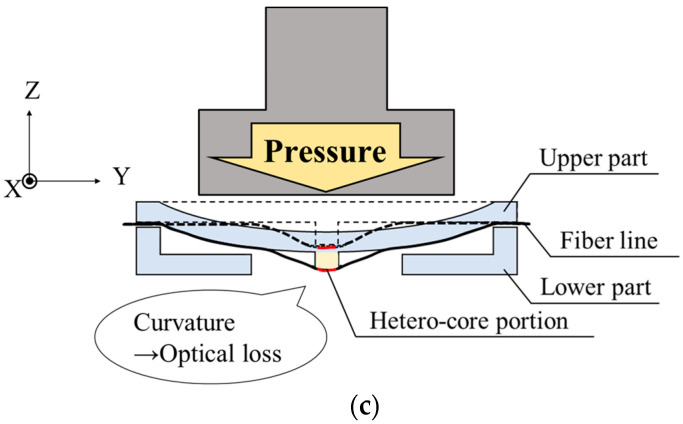
Pressure sensor using hetero-core fiber optics based on conversion mechanism from the pressure to the bending on the hetero-core portion: (**a**) structure of the pressure sensor using hetero-core fiber optics; (**b**) the hetero-core fiber-optic pressure sensor; (**c**) cross-section of the pressure sensor before and after pressurization.

Figure 2 shows the structure of the hetero-core fiber-optic pressure sensor. As shown in Figure 2a, the sensor exhibits a conversion mechanism from pressure to the bending of the hetero-core portion. Previously, we reported on the use of hetero-core fiber-optic pressure sensors implanted in beds for detecting body movement during sleep [19]. The subject’s previously developed pressure sensor exhibited its hetero-core fiber optics embedded in a thin 20 mm square box made of silicone rubber, with a thin elongated structure incorporated into its upper segment. The sensor displayed good-quality monotonic characteristics; however, it did end up requiring enhancements to be able to properly monitor the more subtle pressure changes associated with the elevation of laryngeal prominence during swallowing. In order to enhance the sensor’s pressure sensitivity, the thin structure embedded in the sensor was modified to a 2 mm square protrusion incorporated into its surface B of the upper segment. Additionally, to bend the hetero-core portion more efficiently, a 4.0 mm square hole was fabricated in the center of the lower part of the thin square box. Figure 2b shows a photo of the fabricated pressure sensor using the hetero-core fiber optics. Moreover, the sensor had a length of 20 mm, a width of 20 mm, and a height of 4.2 mm. 

Figure 2c shows the cross-section of the devised hetero-core fiber-optic pressure sensor. The upper part was made of silicone rubber. To bend the hetero-core portion, a protrusion was found in the center of the upper part. The lower part was made of UV-cured resin with a square hole in the center to release the bent hetero-core portion and four L-shaped legs for supporting the upper part. Since the upper part was fixed at only the four legs, it mostly curved toward the center when pressure was applied. This curvature of the upper part made the hetero-core portion curved as well. This principle allows pressure changes applied to the upper part to be converted into changes in the curvature of the hetero-core fiber. Therefore, the applied pressure can be measured as optical loss in the hetero-core fiber sensor.

### 2.2. Wearable Swallowing Assessment Device Using the Hetero-Core Fiber-Optic Pressure Sensor

**Figure 3:**
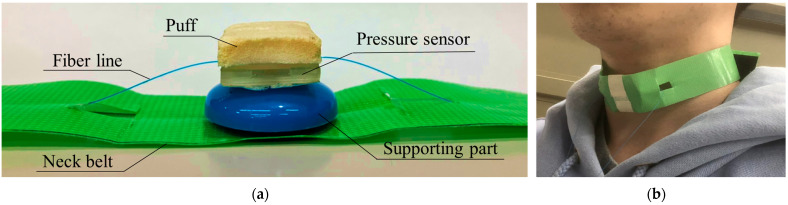
Wearable swallowing test device: (**a**) photograph of the wearable swallowing assessment device; (**b**) appearance when a user attaches the wearable swallowing assessment device.

In order to detect laryngeal movements associated with swallowing as pressure from the skin surface of the laryngeal prominence, we proposed a wearable swallowing assessment device using a fabricated hetero-core fiber-optic pressure sensor attached to a neck belt. Figure 3a provides an illustration of the wearable swallowing assessment device. The device consisted of a puff, a hetero-core fiber-optic pressure sensor, a supporting part, and a belt. The puff was attached to the pressure sensor so that it was comfortable against the skin and reduced pressure upon the larynx. Next, to accurately trace the movement of the displaced laryngeal prominence, the supporting part was configured for the pressure sensor. Figure 3b shows a user’s neck with the attached wearable swallowing assessment device. The belt was used to apply a stable level of pressure between the larynx and the device. The length of the belt was adjustable to the user’s neck size via the use of a hook and a loop fastener.

### 2.3. Experimental Setup

**Figure 4:**
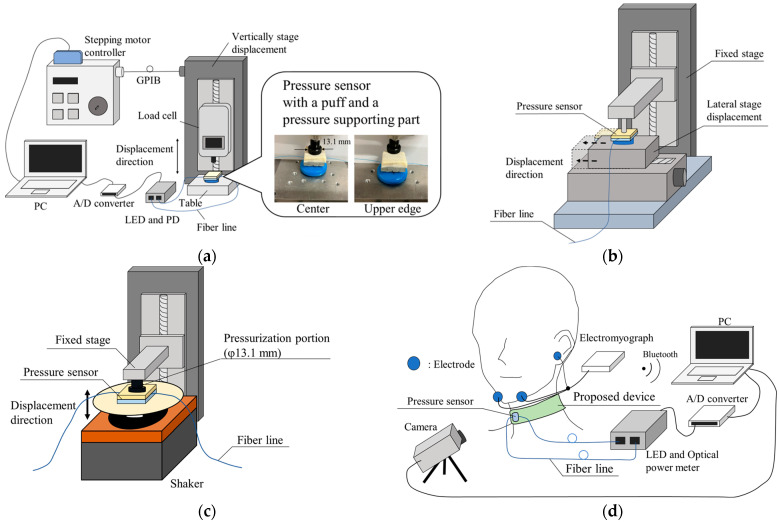
Experimental setup: (**a**) for measuring the pressure characteristics of the pressure sensor; (**b**) for evaluating the sensitivity characteristic with respect to the axial displacement of the applied pressure from the center to the edges of the pressure sensor’s surface; (**c**) for measuring the dynamic characteristics of the pressure sensor; (**d**) for evaluating the response of the proposed wearable swallowing assessment device to laryngeal movement.

Figure 4 shows experimental setups for the hetero-core fiber-optic pressure sensors for the wearable swallowing assessment device. To evaluate the pressure sensors as per their integration within the wearable swallowing assessment device, our team employed an optical power measurement system (iLineBox1S, Core System Japan Co. Ltd., Tokyo, Japan) consisting of an LED with a 1.31 μm wavelength and a photodiode with a light intensity-based measurement protocol. This equipment array resulted in an output analog voltage corresponding to the transmitted light intensity through the hetero-core fiber-optic sensor, which was collected on a PC via an A/D converter (NI USB6008) with a sampling rate of 66 Hz. The evaluation of the hetero-core fiber sensor was based on the pressure input change converted to optical loss in dB. The optical loss was calculated from the output voltage ratio of the optical power measurement system, instead of measuring the pressure input change.

Figure 4a shows the experimental setup for evaluating the pressure characteristic. The hetero-core fiber-optic pressure sensor was set on a table, of which the center and side-edge regions were loaded by the tip, whose diameter was 13.1 mm, of the load cell’s bar. In addition to assessing the effect on the puff and the supporting part, pressure characteristics were likewise independently conducted without the puff and supporting part. To pressurize the sensor by pushing the tip of the load cell, the cell was ultimately controlled by the displacement stage. Next, the load cell (ZP-50N, IMADA Co. Ltd., Aichi, Japan) was mounted on a vertical displacement stage (SG SP 26–100, SIGMA KOKI Co. LTD., Tokyo, Japan), controlled by a stepping motor controller (Stepping Motor Drive SHOT-102, SIGMA KOKI Co. Ltd., Tokyo, Japan). Further, the optical loss of the hetero-core fiber-optic pressure sensor and the applied pressure changes were measured simultaneously. 

Figure 4b shows the experimental setup for evaluating the sensitivity characteristics with respect to the axial displacement of the applied pressure from the center to the edges of the pressure sensor’s surface. The stage configuration consisted of two orthogonal stages, i.e., horizontal and vertical displacement stages. The pressurizer, controlled by the vertical displacement stage, provided a constant pressure of about 15 kPa to the pressure sensor installed on the horizontal displacement stage (SG SP 26–100, SIGMA KOKI Co. LTD., Tokyo, Japan). In order to obtain the constant pressure characteristics from the center to the edges of the pressure sensor’s surface, the horizontal displacement stage was moved a maximum of 10 mm in 0.5 mm increments.

Figure 4c shows the experimental setup for an evaluation of dynamic characteristics for the proposed pressure sensor with the puff. The proposed pressure sensor with the puff was placed on the table of a shaker and fixed from the top by a fixed stage via a pressurization portion. The shaker vibrated up and down to apply sinusoidal time-varying pressure to the pressure sensor. The swallowing waveform had a main frequency of about 1 Hz based on the swallowing time, which corresponds to almost half a period of the swallowing waveform, i.e., less than 0.48–0.65 s. Therefore, sinusoidally varying dynamic pressure values were applied at different frequencies from 1 to 3 Hz.

Figure 4d shows the experimental setup for evaluating the response to swallowing using the wearable swallowing assessment device. To evaluate the response of the wearable neck device during the swallowing action, the subjects wore the swallowing assessment device in its normally designated neck location, as well as electrodes near their chins and earlobes (serving as reference sensors) for an electromyograph (EMG). The EMG measured the surface myoelectric potentials of the geniohyoid muscle, which is involved in laryngeal movement. In addition, a video of the experiment was captured and confirmed the correlation between the laryngeal movements and the response of the wearable swallowing assessment device.

To detect the elevation of the laryngeal prominence using the pressure sensor, the pressure sensor was fixed by the neck belt to ensure that the area of highest pressure sensitivity was in contact with the laryngeal prominence. Due to the sensitivity difference between the center region and edged region of the pressure sensor, positioning in such a fashion initially enabled the pressure change to be easily detected with the elevation of laryngeal prominence. 

The proposed wearable swallowing assessment device was evaluated based on multiple subject testing trials. Sixteen healthy male subjects and one female subject participated in our experiments, and male subjects were divided into four separate age groups. The subjects were seated in chairs with the neck-wearable swallowing devices attached to their larynxes. The tester instructed the subjects to stay absolutely motionless during the swallowing assessment; however, they were not physically restrained in any capacity in order to ensure that uninhibited swallowing took place. The subjects each received 3 mL of water in their mouths and held it for 10 s. This action enabled us to distinguish between the action of holding water in the mouth and the swallowing movement. Ten seconds after holding the water in their mouths, the subjects then swallowed it as instructed by the tester. The swallowing assessments were conducted repeatedly, for a total of nine separate trials per individual subject. 

We provided a necessary explanation for the experiment itself and the details of its planned scope; all of the subjects then signed an informed consent document prior to it being carried out. This study was approved by the SOKA University Institutional Review Board for Human Research.

## 3. Results

### 3.1. Characteristics of the Hetero-Core Fiber-Optic Pressure Sensor

**Figure 5:**
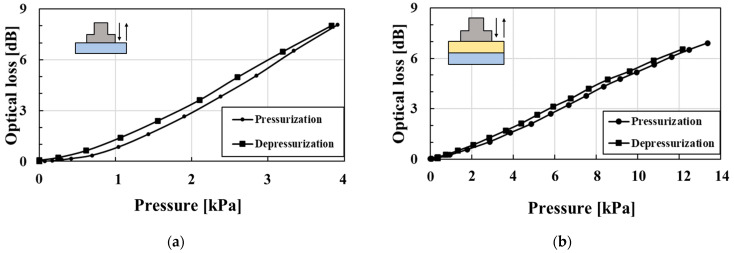

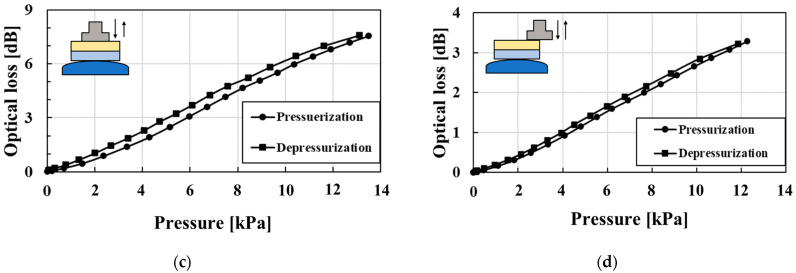
Characteristics of the hetero-core fiber-optic pressure sensor: (**a**) without the puff and the supporting part; (**b**) with the puff; (**c**) with the puff and the supporting part pressurized on the center; (**d**) with a puff and a supporting part pressurized on edge.

Figure 5 shows characteristics of the hetero-core fiber-optic pressure sensor and the resulting optical loss change associated with its use. To comprehensively evaluate the effect on the puff and the supporting part, as shown in Figure 5a–c, respectively, we tested the characteristics of the sensor: (1) without both the puff and the supporting part; (2) with the puff and without the supporting part; and (3) with both the puff and the supporting part. As shown in all of Figure 5a–c, the pressure characteristics indicated monotonic and linear optical loss changes with pressure changes. The sensitivities and R-square values for pressurization and depressurization are shown in Table 1. Since it is not necessary to measure the applied pressure from the larynx due to swallowing, the pressure sensitivity values are unnecessary for the swallowing assessment. We evaluated the sensitivity as the performance of the pressure sensor. The sensitivity of only the fiber-optic pressure sensor shown in Figure 5a was much larger than our previously reported hetero-core fiber-optic pressure sensor for respiration monitoring [19]. Therefore, the fiber-optic pressure sensor for this study was well devised to easily detect the weaker pressure change at the neck compared with the larger-scale pressure change between an entire body and a bed. Compared with the sensitivity of the pressure sensor with and without the puff, as shown in Table 1, the puff reduced the pressure sensitivity; however, the pressure sensor with the puff continually displayed a high degree of linearity. Conversely, the sensitivities with and without the supporting part were very close in value, as shown in Table 1. Therefore, adding the supporting part ultimately did not affect the pressure characteristics of the sensor. When detecting movement with this wearable swallowing assessment device during swallowing, the larynx moves through the surface of the pressure sensor, and the assumption exists that the pressurized portion moves within the sensor surface. Since a sensitivity difference might be present within the surface of the pressure sensor, we evaluated the characteristics of the pressure applied to the edge of the surface, as shown in Figure 5d. Although the sensitivity decreased from 0.592 dB/kPa to 0.274 dB/kPa in pressurization and from 0.606 dB/kPa to 0.280 dB/kPa in depressurization while undergoing pressurizing at the center and at the edge, respectively, the pressure could be detected over the entire surface of the sensor. Focusing on the pressure characteristics at pressurization and at depressurization, hysteresis values of 7.04%, 4.67%, 7.40%, and 4.34% were found, as shown in Figure 5a–d. Adding the puff and the supporting part did not affect any degrees of hysteresis.

**Figure 6:**
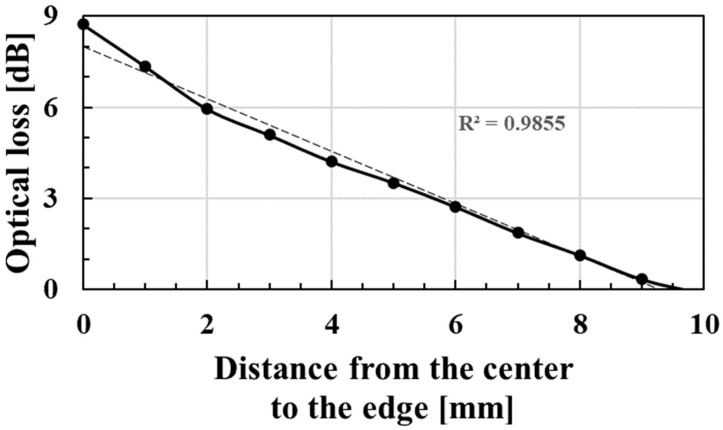
The sensitivity characteristic of the hetero-core fiber-optic pressure sensor with the puff and the supporting part to the axial displacement of the applied pressure from the center to the edges of the pressure sensor’s surface.

Figure 6 shows the sensitivity characteristics to the axial displacement of the applied pressure from the center to the edges of the sensor surface. As shown in Figure 6, the optical loss monotonically decreased as the pressure position moved away from the sensor’s center. This swallowing device utilized the sensitivity difference over the surface of the sensor. If the sensitivity is constant over the surface, then the axial displacement of applied pressure, which corresponds to larynx movement, has difficulty detecting the optical loss change in the swallowing action. Although adopting a multi-sensor array would improve the performance of the localization of applied pressure, the proposed device consisting of even a single sensor can detect swallowing movements due to the sensitivity difference over the surface.

**Figure 7:**
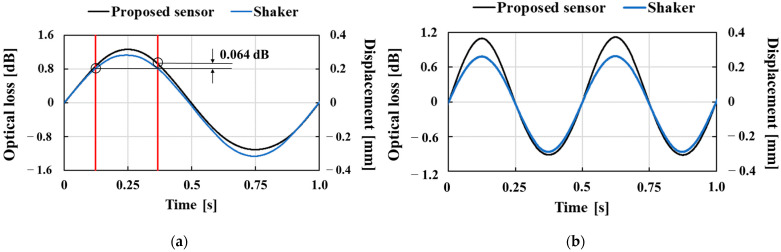

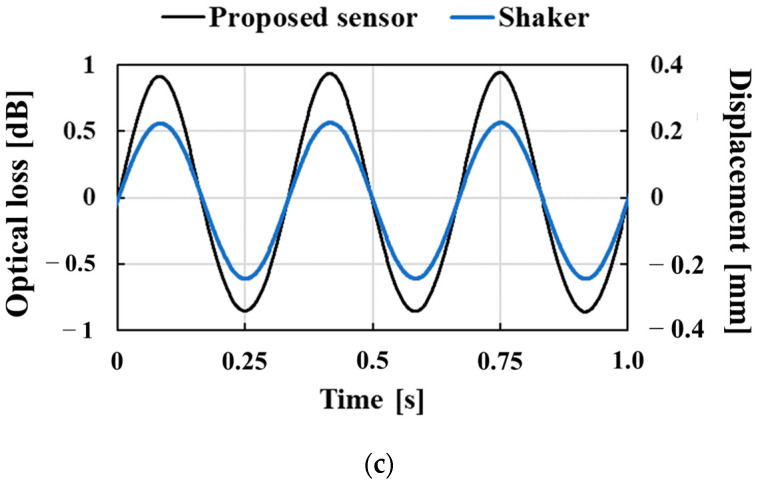
Dynamic characteristics of the hetero-core fiber-optic pressure sensor with the puff applied to sinusoidally time-varying pressure at frequency: (**a**) 1, (**b**) 2, and (**c**) 3 Hz.

Figure 7 shows the proposed pressure sensor’s dynamic characteristics applied to sinusoidal time-varying pressure at frequencies of 1, 2, and 3 Hz. As shown in Figure 7, the optical loss almost followed the dynamic sinusoidal variation in the applied pressure. However, the pressure sensor response showed a slight distortion from the sinusoidal waveform. At a dynamic frequency of 1 Hz, the optical losses at a displacement of 0.20 mm corresponding to a phase of 1/4π were 0.86 and 0.93 dB for the pressurization and depressurization, with the depressurized condition being about 7.5% larger because of the hysteresis of the pressure sensor during pressurization and depressurization. Due to the hysteresis error in the dynamic response, there was a time delay of 0.011 s to reach 0.86 dB during depressurization. Since the swallowing time was roughly in the range of 0.48–0.65 s, the swallowing time measurement was considered to have an error of about 1.7–2.3% due to the hysteresis error.

### 3.2. Evaluation of the Neck-Wearable Swallowing Device

**Figure 8:**
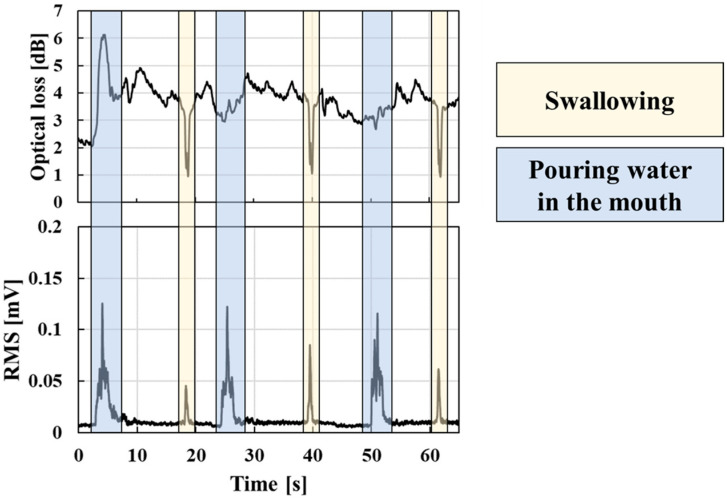
Responses of the wearable swallowing assessment device and EMG during swallowing.

Figure 8 shows a typical response of the wearable swallowing assessment device and the EMG for a male subject in his 30s. The optical loss in the swallowing tests refers to the loss induced before the subject donned the wearable device. As shown in Figure 8, the EMG responded both during swallowing and putting water into the mouth; in contrast, the wearable swallowing assessment device induced an optical loss change only from the swallowing action. The swallowing assessment device could detect a pressure change due to laryngeal movement, while the EMG could detect a muscle voltage change in the neck. Therefore, the experimental results in Figure 8 show that the wearable neck device only captures laryngeal movements because neck muscles can move without laryngeal movement while putting water into the mouth.

**Figure 9:**
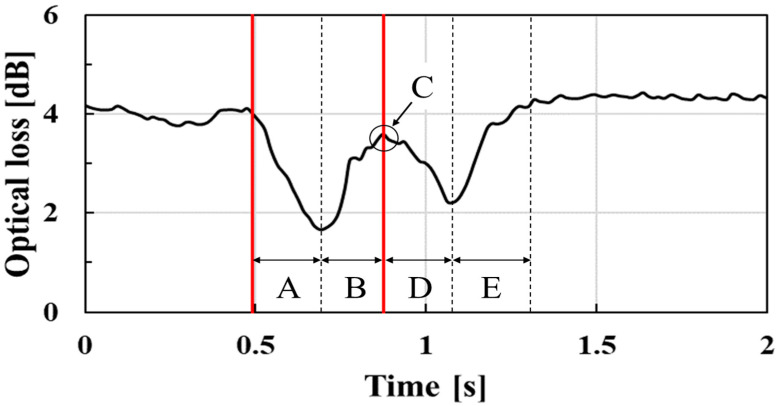
Response by laryngeal movement during swallowing.

We evaluated the relationship between the experimentally obtained waveform and actual laryngeal movement. Laryngeal movement consists of five phases (A–E) of movement as shown in Figure 9: (A) upward movement, (B) upward and forward movement, (C) stationary at the top elevation position, (D) downward and backward movement, and (E) downward movement [8]. The video taken during the swallowing tests found that the first valley of the optical loss indicated in phases (A) and (B) in Figure 9 occurred during laryngeal elevation. Conversely, the second valley of the optical loss indicated in phases (D) and (E) in Figure 9 occurred during laryngeal descent. In phase (A), the optical loss decreased as the laryngeal prominence moved upward because the pressurized position of the laryngeal prominence was away from the center of the pressure sensor surface, in which the pressure sensitivity was the highest. After that, in phase (B), since forward movement was added to the upward movement, the laryngeal prominence additionally pressurized the wearable swallowing assessment device, increasing the optical loss. Optical loss reached its peak phase (C) when laryngeal elevation was finished and backward movement began. Subsequently, during phase (D), the optical loss began to decrease again because backward movement reduced the pressure applied to the swallowing assessment device. Finally, in phase (E), the optical loss once again increased because the pressurized position of the laryngeal prominence was close to the center of the pressure sensor surface as the laryngeal prominence returned to its original position.

The upward movement of “elevation” is additionally classified into two separate stages based on the velocity of laryngeal movement. Natural aging is known to protract the required time of the first elevation stage [8]. Therefore, measuring the time of the first elevation stage enabled us to evaluate the aging effects upon swallowing functionality. Discerning only the first elevation stage (with its different movement velocities) versus the second elevation stage is often difficult given the optical loss temporal waveform obtained by the swallowing assessment device. Due to the sensitivity differences within the sensor’s surface, the device’s sensitivity was constantly changing during elevation. Since the time of the first elevation stage was included in phases (A) and (B), we therefore focused on the time between phases (A) and (B), indicated by the two red lines in Figure 9, as an index for evaluating the swallowing function.

To measure the time of phases (A) and (B), we discerned the time of the first valley in the temporal waveform based on the time derivative, resulting in the determination of the inflection points within the temporal waveform. We set the thresholds to start when the derivative was below −2 dB/s and to end when it was below 2 dB/s, after the start of the swallowing time measurement. This threshold was set as a criterion to exceed the variation in time derivative values without swallowing actions. The temporal response in the optical loss might exceed this threshold without swallowing movements. In such cases, if after the response exceeds the threshold, researchers found that the subject can no longer continue to exceed the threshold within 0.15 s, which is half the typical elevation first-stage time, and the patient is judged not to be in a swallowing motion. Then, the time between phases (A) and (B) was defined as the “swallowing time”. In the proposed device in this paper, only the sensor part was neck-wearable, while the measurement and data analysis parts were desktop systems. Low power consumption and low computational data volume are therefore required for a wearable device that includes a measurement system and data analysis unit. The hetero-core optical fiber has the potential to be a wearable device because it can use an LED light source and detect swallowing waveforms with a data acquisition rate at 66 Hz.

## 4. Discussion

**Figure 10:**
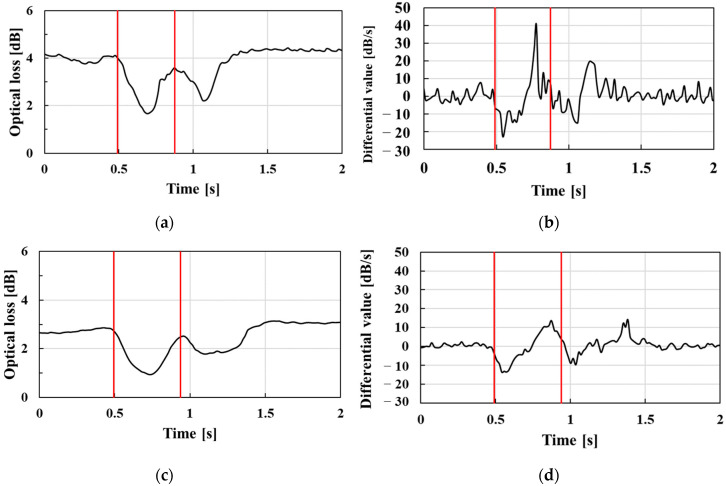

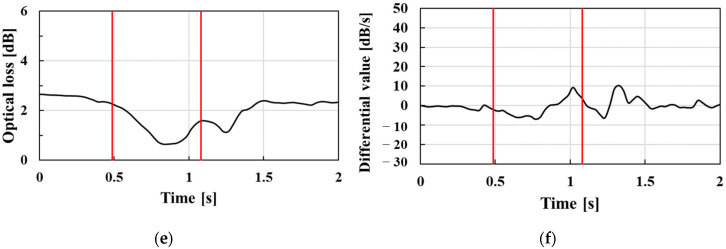
Sensor response with laryngeal movements for different age groups: (**a**) optical loss values for males in their 30s; (**b**) differential values of males in their 30s; (**c**) optical loss values for males in their 60s; (**d**) differential values of males in their 60s; (**e**) optical loss values of female subjects; (**f**) differential values of female subjects.

Figure 10 shows typical temporal waveforms and their derivative profiles obtained by the wearable swallowing assessment device during swallowing for male groups (ranging between 30 and 60 years old) and the female group (in their 20s). Regarding the temporal swallowing waveforms, Figure 10a,c show that the wearable swallowing assessment device could detect two valleys based on the swallowing movements for different age groups. Focusing on the time derivative profiles shown in Figure 10b,d, the swallowing time could be appropriately picked up based on the thresholds shown as the two red lines, compared to the valley period in their temporal waveforms. The typical swallowing time for the patients in their 60s (0.65 s) shown in Figure 10d was 0.17 s longer than for the patients in their 30s (0.48 s). However, since swallowing time strongly depends on a subject’s physique and musculature, which would exhibit deviations within the same age group, we ultimately performed this assessment on multiple subjects for each age group. Furthermore, as shown in Figure 10e, a waveform which exhibited two peaks was obtained in the female case. Only one female subject was included for the feasibility test. Therefore, an evaluation of the swallowing function via the wearable swallowing assessment device may be effective for the female subject. In order to indicate its effectiveness for women, evaluations should be conducted for each age group as well as for male subject in the future.

In Figure 10, a difference in baseline values among three subjects can be observed. The baseline values depended on the overhang of the laryngeal prominence, which changed the degree of neck tilt and the development of laryngeal prominence. Therefore, there were different baselines in subjects. The proposed sensor has a linear pressure characteristic, so its sensitivity does not change even if the initial pressure changes.

**Figure 11:**
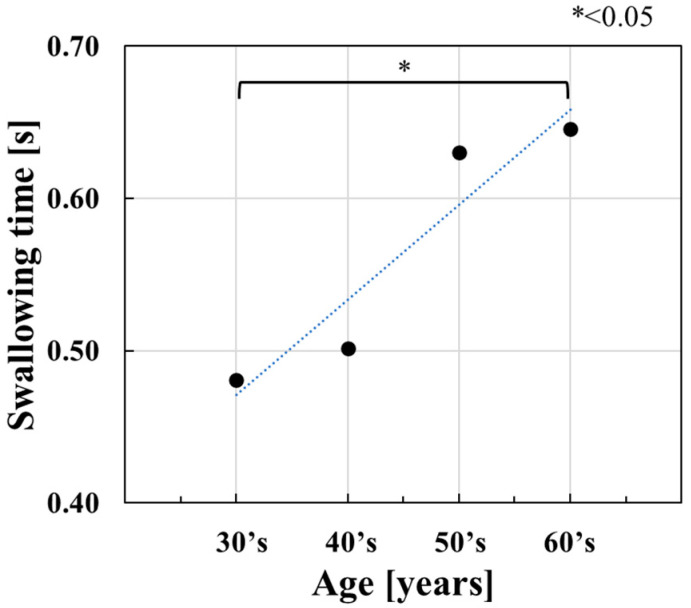
Change in swallowing times versus age in male subjects.

Figure 11 shows the average swallowing times for the different age groups evaluated in this study. Table 2 shows the average age, the number of participants, and the average of swallowing times for each age group. The average swallowing time indicates a positive correlation relative to a group’s age. This characteristic consistently agreed with the increase in the actual time required for the first phase of laryngeal elevation with age [8]. Therefore, the increase in the average swallowing time shows the aging effect.

A one-way analysis of variance and the Tukey–Cramer method were performed for the four distinct age groups utilized for this study. Researchers found a significant difference between the patients in their 30s and 60s (*p* < 0.05). This significant difference was confirmed by the actual extension of time required for laryngeal movement among the distinct groups. 

To analyze whether significant differences exist in swallowing times within each age group, a one-way analysis of variance was performed for each subject within each group. Based on a one-way analysis of variance, the *p*-value, which is used to evaluate the validity of the null hypothesis that all the means are the same, was calculated, resulting in *p* < 0.05 for the patients in their 50s and *p* < 0.01 for all other groups. The results show that there are significant differences in swallowing times among subjects in all age groups.

**Figure 12:**
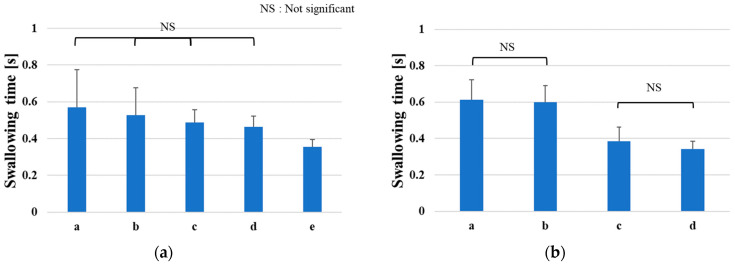

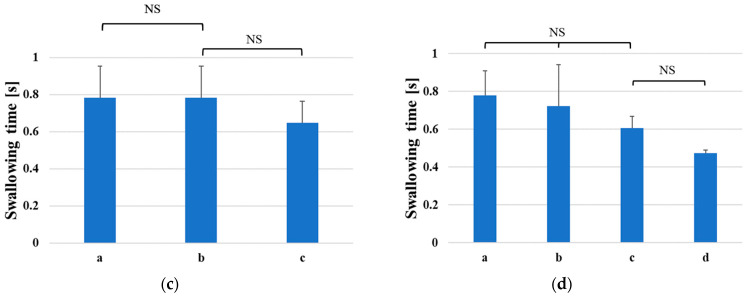
Significant differences in swallowing times between male subjects: (**a**) 30s; (**b**) 40s; (**c**) 50s; (**d**) 60s.

Furthermore, the Tukey–Cramer method was used to examine any significant differences in swallowing times among individuals in each age group. Figure 12 shows the swallowing time and classified results for individual significant differences within each group. If no significant differences are found among individuals, the subjects with the same NS can be classified within the same group regarding the swallowing function. This feature allows users to know whether their swallowing time is longer or shorter than that of the same age group. For example, focusing on the patients in their 40s, as shown in Figure 12b, the four subjects could be classified into two separate subgroups, which means that subjects a and b and c and d belong to the longer and shorter swallowing time groups, respectively, corresponding to a low and high level of swallowing functionality.

## 5. Conclusions

Our research team developed a state-of-the-art neck-wearable swallowing assessment device using a hetero-core fiber-optic pressure sensor technology for detecting laryngeal movement during swallowing. Experimentally, we obtained the characteristics of the subject sensor system via the use of the puff and the supporting part. Its sensitivity and R-square values were measured at 0.592 dB/kPa and 0.995, respectively, during pressurization, and at 0.606 dB/kPa and 0.997, respectively, during depressurization. Moreover, a hysteresis difference of 7.40% was determined for the system. The proposed pressure sensor affords adequate comfortability to the skin of the neck and exhibits high sensitivity and linearity within a pressure domain of 14 kPa. System sensitivity levels were thus better than our team’s previous hetero-core fiber-optic pressure sensor design used for detecting respiration during sleep. Additionally, since the proposed sensor had keen pressure sensitivity over the entire surface of the sensor, the wearable swallowing assessment device was successfully able to track pressure changes induced by laryngeal movements during swallowing.

Sixteen male subjects and one female subject were evaluated using the proposed wearable swallowing assessment device among various ages, ranging from their 30s to their 60s. For all subjects, two valleys in the obtained swallowing waveforms could be observed, which means that the proposed device is able to accurately detect laryngeal movement. Additionally, by successfully measuring the time of the first valley in an acquired waveform, the ability to predictively extend an analogous time interval due to aging was ultimately confirmed. One limitation of this study is that the pressure sensor has a certain degree of stiffness, which limits the ability to reduce the sense of restraint against the neck. Therefore, it is thought that the development of the proposed wearable swallowing assessment devices using flexible materials may be required to reduce this sense of restraint. Finally, the proposed wearable swallowing assessment device is user-friendly at bedside by non-healthcare professionals and patients alike, and it would endeavor to provide an invaluable non-invasive monitoring utility for societies that continue to experience ever-increasing longevities.

## Figures and Tables

**Table 1:** Sensitivities and R-square values of the sensor under each condition.

		Without the Puff and the Supporting Part	With the Puff	With the Puff and the Supporting Part	
Pressurized position		Center			Edge
Pressurization	Sensitivity [dB/kPa]	1.83	0.530	0.592	0.274
	R-square value	0.962	0.996	0.995	0.996
Depressurization	Sensitivity [dB/kPa]	1.90	0.557	0.606	0.280
	R-square value	0.979	0.998	0.997	0.997

**Table 2:** The average age, the number of participants, and the average swallowing times for each age group of male subjects.

**Age Group**	**Average [years]**	**Number of Participants**	**Average of** **Swallowing Time [s]**
30’s	34.4	5	0.48
40’s	48.0	4	0.50
50’s	55.7	3	0.63
60’s	63.3	4	0.65

## Data Availability

Data is contained within the article.
